# Association of Dietary Fiber Intake with All-Cause Mortality and Cardiovascular Disease Mortality: A 10-Year Prospective Cohort Study

**DOI:** 10.3390/nu14153089

**Published:** 2022-07-27

**Authors:** Yu-Jin Kwon, Hye-Sun Lee, Goeun Park, Hyung-Mi Kim, Ji-Won Lee

**Affiliations:** 1 Department of Family Medicine, Yongin Severance Hospital, Yonsei University College of Medicine, Seoul 16995, Korea; digda3@yuhs.ac; 2 Biostatistics Collaboration Unit, Department of Research Affairs, Yonsei University College of Medicine, Seoul 03277, Korea; hslee1@yuhs.ac; 3 Biomedical Statistics Center, Research Institute for Future Medicine, Samsung Medical Center, Seoul 06351, Korea; say.goeun@gmail.com; 4 Department of Food and Nutrition, Dongduck Women’s University, Seoul 02748, Korea; veronykim@naver.com; 5 Department of Family Medicine, Severance Hospital, Yonsei University College of Medicine, Seoul 03722, Korea

**Keywords:** dietary fiber, mortality, cardiovascular diseases, cohort study

## Abstract

Although previous studies have established that dietary fiber (DF) intake reduces the total cardiovascular disease (CVD) mortality in general populations, limited studies have been conducted in individuals with pre-existing chronic conditions, especially in Asian countries. We aimed to investigate the association of DF intake with all-cause and CVD mortality in the general population and in the subpopulation with hypertension, diabetes, and dyslipidemia. We examined the relationship between DF intake and all-cause and CVD mortality using the Korean genome and epidemiology study. Diet was assessed using a food-frequency questionnaire at baseline. Cox proportional hazard models were used to estimate the hazard ratio (HR) and 95% confidence intervals (CIs) after adjusting for confounders. During the mean 10.1 years of follow-up, higher DF intake was significantly associated with a lower risk of all-cause mortality after adjusting for confounders (HR and 95% CIs for Q5 vs. Q1: 0.84 (0.76–0.93); *p* < 0.001). DF intake was inversely associated with a lower risk of CVD mortality after adjusting for the same confounders (HR and 95% CIs for Q5 vs. Q1: 0.61 (0.47–0.78); *p* < 0.001). Total DF intake was inversely associated with all-cause and CVD mortality in middle-aged and older adults.

## 1. Introduction

At the global level, non-communicable diseases (NCDs) contribute to 73.4% of total deaths, whereas cardiovascular disease (CVD) is the leading cause of death and disability [1]. In Korea, CVD mortality and hospitalization have steadily increased over the last decade [2]. In addition, a considerable proportion of adults in Korea have multiple CVD risk factors, such as hypertension, dyslipidemia, and type 2 diabetes. In 2018, approximately 12.1 million adults had hypertension, 4.3 million had diabetes, and 8.7 million had hypercholesterolemia [2].

Since a significant portion of CVD is preventable, the importance of adequate prevention strategies has long been emphasized [3]. Dietary intervention is the first-line approach for preventing CVD, and dietary fiber (DF) has been established as a nutritionally important and health-promoting food component [4]. DF is composed of plant substances that include non-digestible carbohydrates and lignin, which resist digestion by human endogenous enzymes [5]. DF is primarily derived from plant-based foods, such as whole grain, seeds, vegetables, and fruits [6]. Various beneficial effects of fibers on serum low-density lipoprotein (LDL) cholesterol, blood pressure, insulin sensitivity, controlling body weight, and chronic inflammation may exert a protective effect on the cardiovascular system [7,8,9,10]. Indeed, previous studies have established that DF intake reduced the total mortality and CVD mortality in healthy populations [8,11] Accumulating observational studies indicates that DF is inversely associated with risk of hypertension, diabetes, dyslipidemia, peripheral vascular disease, and coronary heart disease [12,13,14].

However, most studies have been conducted in Western countries, and only a limited number of prospective cohort studies on the Asian population have been published [11,15]. Dietary habits and cultures differ by ethnicity, regions, and countries; hence, the main sources of DF could also be different for various countries and subpopulations [11,15,16]. Moreover, few studies have analyzed the effect of DF intake on total and CVD mortality in individuals with pre-existing chronic conditions, which included hypertension, type 2 diabetes, and dyslipidemia [12]. Therefore, we aimed to investigate the association of DF intake with all-cause and CVD mortality in middle-aged and older Korean adults. Further, we analyzed the effect of DF intake on all-cause and CVD mortality in the subpopulation with hypertension, diabetes, and dyslipidemia.

## 2. Materials and Methods

### 2.1. Study Population

We analyzed baseline survey data from the Korean genome and epidemiology study (KoGES),_Ansan–Ansung study (2001–2002), KoGES_health examinee study (2004–2013), and the KoGES_cardiovascular disease association study (2005–2011), which were large-scale, longitudinal, and prospective cohort studies that investigated the risk factors for NCDs [17]. This study included 211,571 adults aged 40 years and older, who had lived in urban and rural areas for ≥6 months. The survey began in the year 2001 and is presently ongoing.

**Figure 1:**
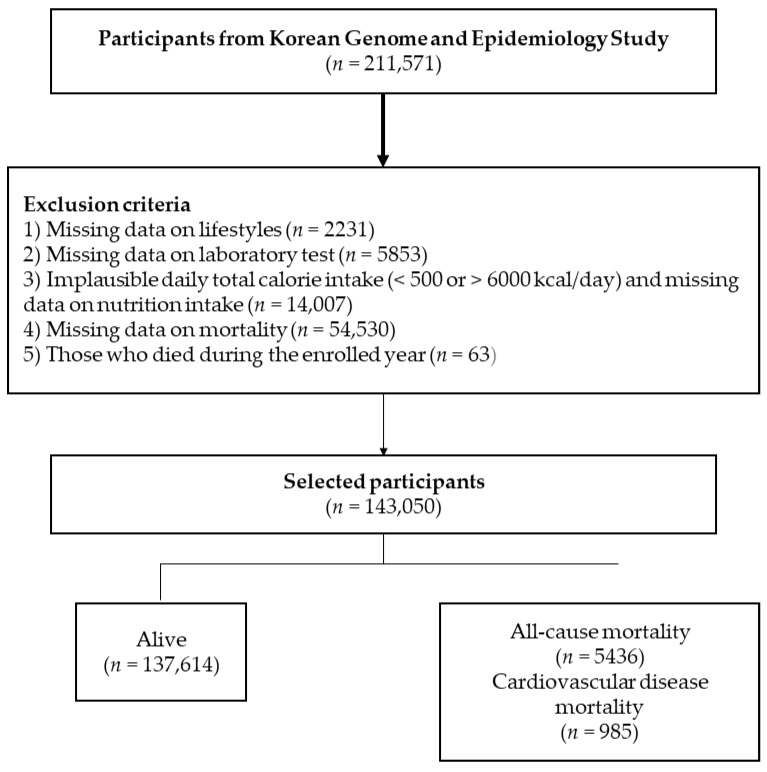
Selection process of study population.

Figure 1 shows the participant selection process. From the 211,571 participants included in the baseline survey, participants were excluded for further study based on the following criteria: (1) lack of data on age and other lifestyle factors (*n* = 2231); (2) missing data on laboratory tests (*n* = 5853); (3) missing dietary information and total calorie intake, <500 kcal/day or >6000 kcal/day (*n* = 14,007); (3) missing data regarding death information (*n* = 54,530); and (4) death during the enrolled year (*n* = 63). Finally, we included a total of 143,050 participants. All participants provided written informed consent. The study protocol conformed to the ethical guidelines of the 1975 Declaration of Helsinki. This study was approved by the IRB of Yongin Severance Hospital.

### 2.2. Dietary Assessment

A food frequency questionnaire (FFQ) was used to assess the dietary intake. The FFQ of the KoGES-Ansan-Ansung baseline study contains 103 food items, while those of the KoGES-HEXA and KoGES_CAVAS contain 106 food items.

The FFQ evaluated how often the participants consumed each food item (never or seldom, once a month, two to three times per month, one to two times per week, three to four times per week, five to six times per week, once a day, twice per day, or thrice per day) and the amount of a particular food that they consumed in each meal (a half serving, one serving, or two or more servings) during the past 1 year. DF intake and other nutrients were calculated using the FFQ. We recorded the DF intake as g/day and divided it into quintiles (Q1 to Q5). The FFQ used in this study was available on the following website: http://www.cdc.go.kr/contents.es?mid=a40504100100 (accessed on 27 December 2021).

### 2.3. Covariates

Trained medical staff performed all health examination procedures according to the standardized protocols published by KoGES. Measurements for height were obtained to the nearest 0.1 cm, and body mass index (BMI) was calculated using the height and weight. Blood pressure was measured after 5minutes resting in a sitting position. Blood tests were conducted after 8 h fasting. Serum total cholesterol, high-density lipoprotein (HDL), triglyceride, and glucose levels were enzymatically analyzed using a Chemistry Analyzer (Hitachi 7600, Tokyo, Japan from August 2002 and ADVIA 1650, Siemens, Tarrytown, NY from September 2002). A self-questionnaire was used to assess the status of smoking, alcohol drinking and exercise. Smokers were classified as current smokers, former smokers, and non-smokers. People consuming alcohol were classified as current drinkers, former drinkers, and non-drinkers. A person who regularly exercised enough to sweat was defined as a regular exerciser. A person with a systolic blood pressure ≥140 mmHg, diastolic blood pressure ≥90 mmHg, or diagnosis by physician was corresponded to hypertension. A person with fasting plasma glucose level ≥126 mg/dL, glycosylated hemoglobin ≥6.5%, or diagnosis by physician was corresponded to diabetes. A person with total cholesterol level ≥200 mg/dL, triglyceride ≥150 mg/dL, or diagnosis by physician was corresponded to dyslipidemia.

### 2.4. Study Outcomes

The primary outcome was all-cause mortality. Mortality outcomes were ascertained by the death records provided by Korea National Statistical Office. Participant deaths were tracked from the initial assessment up to 2019. All-cause mortality included all the specified and unknown causes of death. CVD mortality included deaths from International Classification of Diseases-10 codes I00-I99.

### 2.5. Statistical Analyses

Continuous data are presented as mean ± standard deviation (SD) and categorical data are presented as number (%). Among-group comparisons of continuous and categorical variables were performed using analysis of variance and the chi-square test, respectively. Cox proportional hazard regression model was used to calculate the hazard ratio (HR) and determine the association between DF intake and all-cause mortality and CVD mortality. Kaplan–Meier curves with the log-rank test were used to calculated the cumulative all-cause mortality and CVD mortality, according to the DF intake quintiles. The warranty period was defined as the time taken to reach the cumulative mortality rate of >0.5% for each group. If the 0.5% threshold was not met, the value was expressed as the time of the last follow-up. Incidence per 1000 person-years was calculated for each group. We calculated the HR and 95% confidence interval (CI) for all-cause mortality and CVD mortality, based on the DF intake quintile groups. In model 1, we adjusted for age, sex, body mass index, smoking, alcohol intake, exercise, and total calorie. In model 2, we further adjusted for hypertension, diabetes, and dyslipidemia. We conducted subgroup analysis according to the presence of hypertension, diabetes, and dyslipidemia. SAS statistical software (version 9.4; SAS Institute Inc., Cary, NC, USA) and R (Version 4.0.3; R Foundation for Statistical Computing, Vienna, Austria) were used for statistical analyses. Statistical significance was set at *p* ≤ 0.05.

## 3. Results

During the median (min, max) 10.1 years (0.2, 15.9) follow-up period, 5436 cases of all-cause mortality and 985 cases of CVD mortality were noted. Among the total 143,050 participants, the mean age ± SD was 53.9 ± 8.7 years, and the proportion of men was 35.6%.

Table 1 shows the baseline characteristics of study population, according to the DF intake quintiles. Men (*p* < 0.001), older adults (*p* < 0.001), and current smokers (*p* < 0.001) were less likely to have a higher DF intake. Participants with the highest DF intake had lower serum glucose level (*p* < 0.001) and triglyceride level (*p* = 0.022). Moreover, participants with the highest DF intake had higher BMI (*p* < 0.001), waist circumference (*p* < 0.001), and serum HDL-C level (*p* = 0.001) and were more likely to regularly exercise (*p* < 0.001) and live in urban areas (*p* < 0.001). Regarding nutritional intake, participants with the highest DF intake consumed higher total energy and other nutrients, while they consumed less carbohydrate proportion.

**Figure 2:**
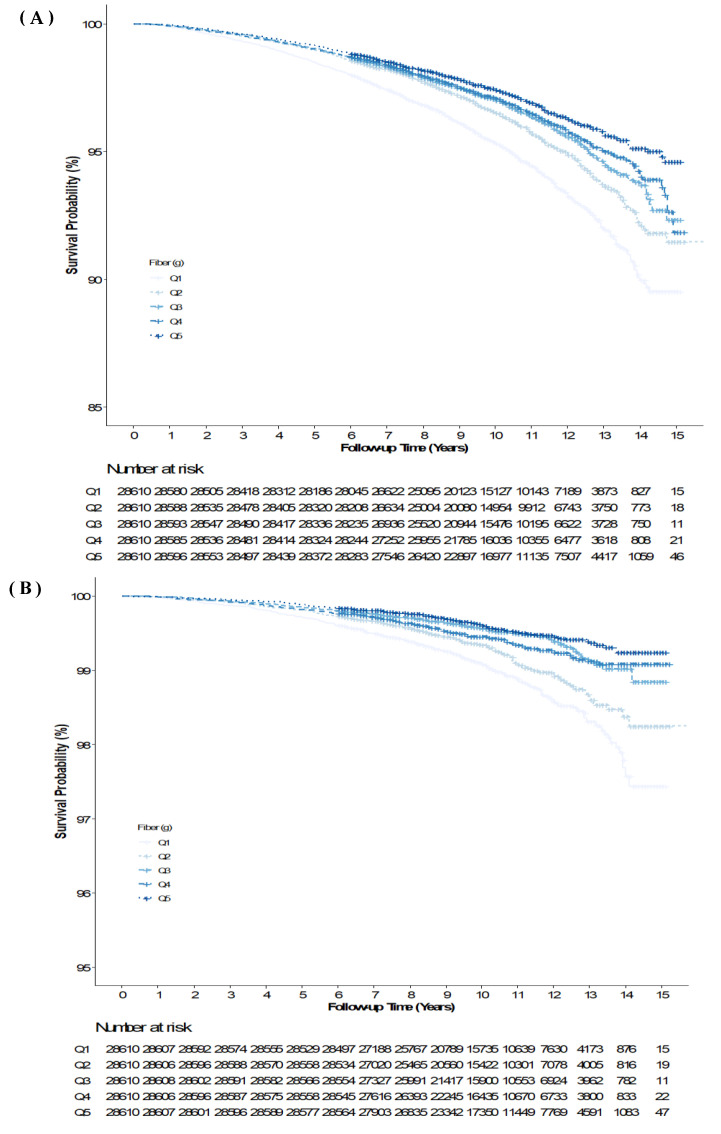
Kaplan–Meier curves displaying the estimated survival probability for fiber intake quintile groups. (**A**) All-cause mortality across the dietary fiber quintiles. (**B**) Cardiovascular disease mortality across the dietary fiber quintiles.

Figure 2 presents the cumulative all-cause mortality and CVD mortality of DF intake quintiles as Kaplan–Meier curves. The lowest DF intake group showed significantly the highest cumulative all-cause mortality, followed by Q2, Q3, Q4, and Q5 (log-rank test, *p* < 0.001). There was a similar trend in the cumulative CVD mortality event curves among the DF intake quintile groups (log-rank test, *p* < 0.001).

Table 2 shows the warranty period of all-cause mortality and CVD mortality for all the DF intake quintiles. All-cause mortality was associated with the longest 0.5% warranty period of 3.50 years for participants in the highest DF intake group. CVD mortality was associated with the longest 0.5% warranty period of 11.17 years for those in the highest DF intake group. The observed durations of the warranty period for both all-cause mortality and CVD mortality was the shortest in Q1 of DF intake. The incidence rate for all-cause mortality was lowest for participants with the highest DF intake (2.87 per 1000 person-years; 95% CI, 2.25–3.49). The incidence rate for CVD mortality was also the lowest for participants with the highest DF intake (0.42 per 1000 person-years; 95% CI, 0.18–0.66). The absolute numbers of all-cause mortality were 1511 in Q1, 1126 in Q2, 990 in Q3, 951 in Q4, and 858 in Q5. The number of CVD mortality was the lowest in Q5 (*n* = 127).

Table 3 shows the multiple Cox proportional hazard regression analysis for all-cause mortality and CVD mortality. The HR (95% CI) for all-cause mortality in Q5 with reference to Q1 was 0.84 (0.76–0.93, *p* < 0.001) after adjusting for age, sex, BMI, smoking, alcohol intake, exercise, total calories, hypertension, diabetes, and dyslipidemia. The HR (95% CI) for CVD mortality in Q5 with reference to Q1 was 0.61 (0.47–0.78, *p* < 0.001), after adjusting for the same confounders.

**Figure 3:**
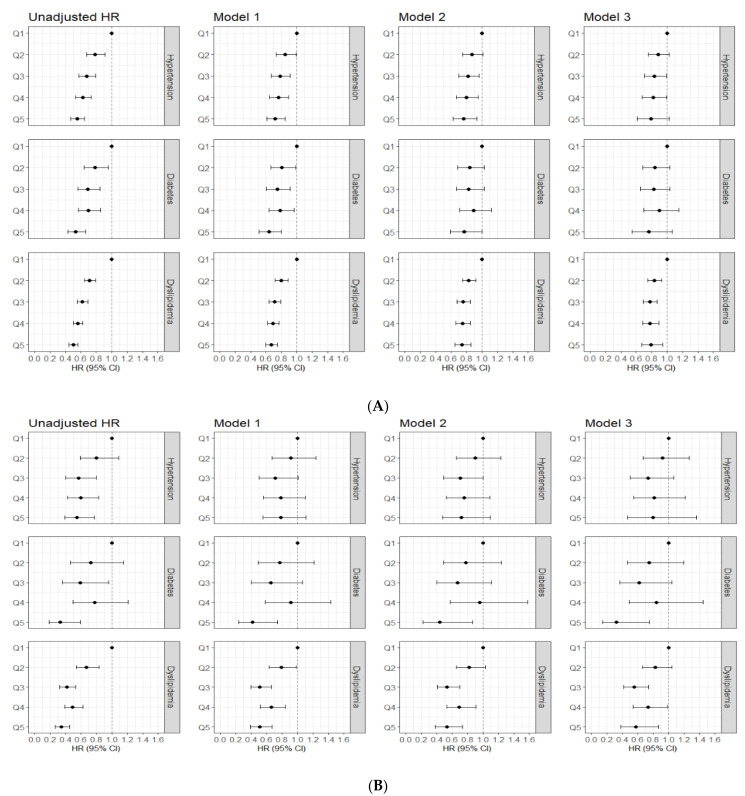
Association between dietary fiber intake and all-cause mortality and cardiovascular disease mortality according to hypertension, diabetes, and dyslipidemia. (**A**) All-cause mortality, (**B**) Cardiovascular disease mortality. HR: hazard ratio; CI: confidence interval.

Figure 3 depicts the HR and 95% CI for all-cause mortality and CVD mortality, according to DF intake by participants with hypertension, diabetes, and dyslipidemia. In the baseline survey, there were 24,407 hypertension cases, 10,364 diabetes cases, and 81,219 dyslipidemia cases. During the median follow-up period of 10.3 years, 9.4 years, and 10.2 years, there were 1411 deaths in patients with hypertension, 838 deaths in patients with diabetes, and 3008 deaths in patients with dyslipidemia, respectively. Further, the numbers of CVD deaths were 324 in patients with hypertension, 153 in patients with diabetes, and 591 in patients with dyslipidemia, respectively. The HR (95% CI) for all-cause mortality in Q5 compared with Q1 was 0.55 (0.47–0.65) in patients with hypertension. Similar trends remained after adjusting for confounders. The HR (95% CI) for CVD mortality in Q5 compared with Q1 was 0.55 (0.39–0.77) in patients with hypertension. However, the significant association was attenuated after adjusting for confounders. In patients with diabetes, the HRs (95% CI) for all-cause mortality and CVD mortality in Q5 compared with Q1 were 0.53 (0.43, 0.66) and 0.33 (0.19, 0.59), respectively. However, this significant association between DF intake and total mortality disappeared after adjusting for confounders. Regarding CVD mortality, a significant inverse association between DF intake and CVD mortality was observed after adjusting for confounders. The HRs (95% CIs) for all-cause mortality and CVD mortality were 0.50 (0.49, 0.56) and 0.35 (0.27, 0.45), respectively, in patients with dyslipidemia, and significant association remained after adjusting for confounders. The detailed figures were described in Supplementary Table S1.

## 4. Discussion

In this study, we found that DF intake was significantly inversely associated with all-cause mortality and with CVD mortality in middle-aged and older Korean adults.

Poor dietary habits are associated with many chronic diseases and can be a major contributor to mortality [18]. In contrast, a healthy diet is one of the most promising factors in primary and secondary prevention of NCD [19]. DF, “the seventh nutrient” of the body, is a component of fruits, vegetables, and whole grains, and is non-replaceable to maintain health [20]. DF has anti-inflammatory [10] and antioxidative properties [21] and has been shown to lower blood pressure [7], reduce serum cholesterol [22] and glucose levels [9], improve endothelial function [23], reduce body weight loss, aid favorable changes of gut microbial composition, and reduce the contact time between carcinogens and intestinal mucosal cells by increasing fecal bulking and viscosity [24]. All of these biochemical effects may be potentially related to a lower risk of chronic diseases and mortality.

Prospective studies have shown an inverse association between DF intake and all-cause and CVD mortality [11,15,25,26,27,28,29,30]. The NIH-AARP Diet and Health Study that had an average 9 years follow-up (567,169 participants aged 50–71 years from six states of the U.S.) found that 10 g/day increment of DF consumption reduced total mortality by 12% and 15% in men and women, respectively [25]. The Zutphen Study reported that 10 g/day of fiber increment reduced all-cause mortality by 9% and CVD mortality by 17% [29]. A meta-analysis from the prospective cohort studies reported that DF intake was inversely associated with the risk of CVD and coronary heart diseases [14]. Recently, the Japan Public Health Center-based prospective study with 92,924 participants found that total mortality was reduced by 23% in men and 18% in women for the highest quintile of total fiber intake [11]. Our data were consistent with the results of the previous studies.

Current recommendations for DF intake for adults in most European countries and in the U.S. are 30–35 g/day for men and 25–32 g/day for women [31]. Similarly, the Korean Nutrition Society suggested that sufficient amount of daily fiber intake for Korean adults is 25 g/day for men and 20 g/day for women regardless of age [32]. However, the global consumption of fiber falls below the recommended levels, regardless of the country [31]. In our study, the amounts of DF intake were far below the recommended daily intake in Korea. Nonetheless, we found that for the highest quintile of total fiber intake, all-cause mortality was reduced by 16% and CVD mortality by 39%. Our data suggested that a relatively small increase in fiber intake may offer a public health benefit in reducing all-cause and CVD mortality.

DF reportedly exerts its influence on CVD mortality by decreasing the risk of hypertension, diabetes, and dyslipidemia in different patterns [7,9]. Epidemiologic studies consistently show that increased fiber intake is significantly associated with reduced hypertension risk. Meta-analyses of intervention trials showed that increased fiber intake is more effective in lowering blood pressure in hypertensive individuals than in normotensive individuals [33]. Therefore, increased fiber intake may adjunctively further lower blood pressure in hypertensive individuals. Further, systematic reviews conducted in 2010–2018 reported that diet high in soluble fiber caused at least moderate (i.e., 0.20–0.40 mmol/L) reductions in LDL cholesterol [34]. The bulking effect, viscosity, fermentation, and increased production of short-chain fatty acids are believed to be responsible for the antihyperlipidemic benefits of DFs [24]. Although the blood glucose-lowering effects of DF are inconsistent, three meta-analyses presented a 15–19% reduction in the incidence of developing type 2 diabetes, when participants with the highest intake of total DF to those with the lowest intake were compared [35,36,37]. A few studies have examined the effect of DF intake on mortality in patients who had hypertension, diabetes, or dyslipidemia and have reported inconsistent results [25,30,38]. Patients with hypertension, diabetes, and dyslipidemia have a 2–3 times higher risk of CVD and premature mortality than the general population [39,40]. We analyzed the effect of DF intake on total and CVD mortality in these patients and found that DF intake lowered the risk of all-cause mortality in patients with hypertension and dyslipidemia, and the risk of CVD mortality in patients with diabetes and dyslipidemia. Our findings suggest that low DF may be considered an important modifiable risk factor for decreasing mortality in patients with hypertension, diabetes, and dyslipidemia. However, the significant association between DF intake in patients with hypertension and CVD mortality, and that between DF intake in patients with diabetes and all-cause mortality were attenuated after adjusting for confounders. Although the exact mechanism is not known, different effects of DF subtypes and sources may explain the results to some extent. Vegetables, cereals, and fruits were three major sources of DF for Koreans who obtained approximately 75% of DF from those sources. Kimchi, a traditional fermented food made by salting and fermenting vegetables, was the first major source of DF for Koreans aged over 12 years [41,42]. A negative impression of kimchi is that as it is high in sodium and could be the cause of high blood pressure and metabolic syndrome [43]. Moreover, the sugar content in fruits is generally higher than in vegetables, leading to concerns about its potentially harmful impacts on patients with diabetes [44]. There are inconsistent results about the effects of fruit consumption on risks of death and major complications among people with established diabetes [45,46,47]. Further studies are needed to identify the association between DF and mortality considering the specific dietary type and sources.

Our study had several limitations. First, the FFQ method used in this study has the potential for recall bias, and DF could be underestimated. However, KoGES data are reliable and widely used. Moreover, it is the only available large dataset that contains both information about nutrition assessed by FFQ and mortality status in Korea. Second, we could not measure the overall diet quality due to restricted data, and there could be potential and unmeasured confounders that were not fully considered. However, the sample size used in this study was sufficiently large to detect significant HRs over 90% power (Q1 vs. Q5). Therefore, our study population was large enough to detect meaningful associations. Third, our study could not differentiate subtypes and sources of DF. Since we obtained an integrated dataset using the three KoGES data and not the original FFQ dataset, we could only use the total DF amount calculated by FFQ. Fourth, since our analysis only included middle-aged and older Korean adults, the current findings cannot be generalized to other countries and ethnic groups. Fifth, we could not consider the information about anti-hypertensive, anti-diabetic, and anti-dyslipidemia medications. Finally, we assessed the baseline DF intake, which was not updated during the follow-up period. Thus, baseline exposures might not reflect changes in DF intake over time.

To the best of our knowledge, this is the first study that investigated the association between DF intake and mortality in Korea using the largest population-based dataset over a long duration of follow-up. Furthermore, we comprehensively analyzed the effect of DF intake on total and CVD mortality in individuals with pre-existing hypertension, type 2 diabetes, and dyslipidemia.

## 5. Conclusions

In the current study, the total fiber intake was inversely associated with all-cause and CVD mortality. DF intake lowered the risk of all-cause mortality in patients with hypertension and dyslipidemia and the risk of CVD mortality in patients with diabetes and dyslipidemia. In the view of public health policies, adequate fiber consumption with healthy diet habits should be emphasized for reducing mortality and premature NCDs mortality.

## Figures and Tables

**Table 1:** Baseline characteristics of the cohort according to fiber intake (g/day).

Variables	Q1 (0.37, 3.51)	Q2 (3.51, 4.64)	Q3 (4.64, 5.79)	Q4 (5.79, 7.44)	Q5 (7.44, 52.65)	*p*-Value
N	28,610	28,610	28,610	28,610	28,610	
Sex, men, n (%)	10,033 (35.1)	10,308 (36.0)	10,438 (36.5)	10,315 (36.1)	9850 (34.4)	<0.001
Age, years	54.7 ± 9.4	53.9 ± 8.8	53.8 ± 8.6	53.6 ± 8.4	53.2 ± 8.2	<0.001
BMI, kg/m^2^	23.8 ± 3.0	23.9 ± 2.9	24.0 ± 2.9	24.0 ± 2.9	24.1 ± 2.9	<0.001
WC, cm	81.0 ± 8.9	81.3 ± 8.7	81.4 ± 8.8	81.5 ± 8.7	81.4 ± 8.8	<0.001
SBP, mmHg	122.5 ± 15.7	122.7 ± 15.5	122.8 ± 15.3	122.7 ± 15.3	122.5 ± 15.2	<0.001
DBP, mmHg	76.1 ± 10.2	76.1 ± 10.1	76.2 ± 10.0	76.2 ± 10.0	76.4 ± 10.0	<0.001
Glucose, mg/dL	96.0 ± 21.9	95.9 ± 21.9	95.7 ± 20.6	95.4 ± 21.0	95.2 ± 21.3	<0.001
HbA1c, %	5.72 ± 0.77	5.71 ± 0.75	5.72 ± 0.73	5.72 ± 0.75	5.72 ± 0.74	0.901
TC, mg/dL	197.5 ± 36.1	197.2 ± 35.6	197.3 ± 35.7	197.7 ± 35.6	197.2 ± 35.5	0.392
HDL-C, mg/dL	52.4 ± 13.3	52.6 ± 13.1	52.6 ± 13.0	52.7 ± 13.0	52.9 ± 12.9	0.001
LDL-C (mg/dL)	119.3 ± 32.9	118.9 ± 32.7	119.0 ± 32.8	119.2 ± 32.6	119.0 ± 32.4	0.469
TG, mg/dL	129.9 ± 90.3	130.0 ± 92.4	129.3 ± 91.3	130.1 ± 91.1	127.9 ± 89.4	0.022
Smoking status, n (%)						<0.001
Never smoker	20,428 (71.4)	20,424 (71.4)	20,406 (71.3)	20,525 (71.7)	21,004 (73.4)	
Former smoker	4177 (14.6)	4421 (15.5)	4602 (16.1)	4467 (15.6)	4218 (14.7)	
Current smoker	4005 (14.0)	3765 (13.2)	3602 (12.6)	3618 (12.7)	3388 (11.8)	
Alcohol intake, n (%)						<0.001
Never drinker	14,618 (51.1)	14,355 (50.2)	14,348 (50.2)	14,418 (50.4)	14,633 (51.2)	
Former drinker	1271 (4.4)	1155 (4.0)	1100 (3.8)	1049 (3.7)	1210 (4.2)	
Current drinker	12,721 (44.5)	13,100 (45.8)	13,162 (46.0)	13,143 (45.9)	12,767 (44.6)	
Regular exercise (No)	12,359 (43.2)	13,410 (46.9)	14,468 (50.6)	15,139 (52.9)	16,353 (57.2)	<0.001
HTN, n (%)	4997 (17.5)	4861 (17.0)	4842 (16.9)	4809 (16.8)	4898 (17.1)	0.272
DM	2165 (7.6)	2157 (7.5)	2064 (7.2)	2019 (7.1)	1959 (6.9)	0.003
Dyslipidemia	16,216 (56.7)	16,348 (57.1)	16,228 (56.7)	16,344 (57.1)	16,083 (56.2)	0.147
Residential area, n (%)						<0.001
Urban	23,776 (83.1)	24,648 (86.2)	24,993 (87.4)	25,467 (89.0)	25,472 (89.0)	
Rural	4834 (16.9)	3962 (13.9)	3617 (12.6)	3143 (11.0)	3138 (11.0)	
Total energy, kcal/day	1310.3 ± 344.0	1541.9 ± 356.2	1695.2 ± 377.3	1874.8 ± 415.3	2256.4 ± 630.7	<0.001
Carbohydrate intake, g/day	242.8 ± 64.9	279.7 ± 64.8	304.1 ± 66.3	332.3 ± 71.3	389.4 ± 100.7	<0.001
Carbohydrate (%)	74.2 ± 7.1	72.8 ± 6.7	72.1 ± 6.5	71.2 ± 6.5	69.70± 7.4	<0.001
Fat, g/day	17.4 ± 10.5	22.6 ± 12.0	26.1 ± 13.1	30.3 ± 15.0	40.2 ± 22.9	<0.001
Fat (%)	11.8 ± 5.7	13.01 ± 5.3	13.6 ± 5.1	14.2 ± 5.0	15.4 ± 5.4	<0.001
Protein, g/day	38.9 ± 12.7	49.0 ± 14.1	55.9 ± 15.6	64.4 ± 17.9	84.2 ± 31.3	<0.001
Protein (%)	11.9 ± 2.3	12.8 ± 2.2	13.2 ± 2.2	13.8 ± 2.3	14.9 ± 2.8	<0.001
Sodium, mg/day	1135.9 ± 492.0	1837.7 ± 562.3	2355.9 ± 665.6	2919.9 ± 783.2	4306.2 ± 1666.1	<0.001
Potassium, mg/day	1183.5 ± 392.1	1684.8 ± 377.2	2059.3 ± 410.6	2512.0 ± 474.2	3642.7 ± 1151.1	<0.001
Ca, mg	229.8 ± 125.2	328.7 ± 135.5	402.5 ± 150.7	491.9 ± 168.0	736.1 ± 321.0	<0.001
P, mg	571.8 ± 168.2	731.7 ± 177.5	843.6 ± 197.1	975.3 ± 222.5	1286.9 ± 415.7	<0.001
Fe, mg	5.4 ± 1.6	7.5 ± 1.7	9.1 ± 1.9	11.0 ± 2.3	16.3 ± 6.0	<0.001
Vit. A, R.E	201.4 ± 98.0	316.4 ± 117.5	405.8 ± 139.1	524.9 ± 173.3	920.7 ± 474.9	<0.001
Vit. B1, mg	0.64 ± 0.23	0.82 ± 0.25	0.94 ± 0.26	1.10 ± 0.30	1.47 ± 0.51	<0.001
Vit. B2, mg	0.53 ± 0.22	0.71 ± 0.25	0.84 ± 0.27	0.99 ± 0.30	1.38 ± 0.54	<0.001
Niacin, mg	9.38 ± 3.09	11.92 ± 3.36	13.66 ± 3.68	15.77 ± 4.22	20.80 ± 7.42	<0.001
Vit. C, mg	42.45 ± 20.01	70.18 ± 22.86	92.71 ± 27.07	121.30 ± 33.19	196.14 ± 82.27	<0.001
Zinc, μg	5.38 ± 1.76	6.65 ± 2.05	7.52 ± 2.30	8.59 ± 2.70	11.15 ± 4.90	<0.001
Vit. B6, mg	0.93 ± 0.25	1.24 ± 0.25	1.46 ± 0.28	1.74 ± 0.33	2.47 ± 0.80	<0.001
Folate, μg	98.94 ± 30.53	152.45 ± 28.69	193.63 ± 33.93	243.46 ± 44.54	385.96 ± 146.10	<0.001
Retinol, μg	41.20 ± 38.49	54.77 ± 46.34	63.83 ± 48.57	74.72 ± 54.54	102.65 ± 87.49	<0.001
Carotene, μg	925.6 ± 469.8	1520.1 ± 585.1	1991.6 ± 720.7	2621.8 ± 924.6	4779.4 ± 2648.2	<0.001
Vit. E, mg	4.49 ± 1.75	6.09 ± 2.04	7.31 ± 2.37	8.86 ± 2.77	13.24 ± 5.48	<0.001
Fiber, g	2.7 ± 0.6	4.1 ± 0.3	5.2 ± 0.3	6.5 ± 0.5	10.1 ± 3.0	<0.001

BMI, body mass index; DBP, diastolic blood pressure; DM, diabetes mellitus; HTN, hypertension; HbA1c, hemoglobin A1c; HDL-C, high density lipoprotein cholesterol; LDL-C, low density lipoprotein cholesterol; R.E, retinol equivalents; SBP, systolic blood pressure; TC, total cholesterol; TG, triglycerides; WC, waist circumference.

**Table 2:** Warranty periods for mortality according to fiber intake (g/day).

**All-Cause Mortality**					
**Variables**	**Warranty Period (0.5%)**	**n**	**Person-Time (Years)**	**Events, n**	**Incidence per 1000 Person-Years (95% CI)**
Fiber (g)					
Q1 (0.37, 3.51)	2.42	28,610	289,394.9	1511	5.22 (4.39–6.06)
Q2 (3.51, 4.64)	3.09	28,610	288,648.6	1126	3.90 (3.18–4.62)
Q3 (4.64, 5.79)	3.34	28,610	291,149.0	990	3.40 (2.73–4.08)
Q4 (5.79, 7.44)	3.09	28,610	293,349.0	951	3.24 (2.58–3.90)
Q5 (7.44, 52.65)	3.50	28,610	299,095.9	858	2.87 (2.25–3.49)
**CVD Mortality**					
**Variables**	**Warranty Period (0.5%)**	**n**	**Person-Time (Years)**	**Events, n (%)**	**Incidence per 1000 Person-Years (95% CI)**
Fiber (g)					
Q1 (0.37, 3.51)	6.84	28,610	294,427.0	311	1.06 (0.68–1.43)
Q2 (3.51, 4.64)	8.50	28,610	292,325.6	231	0.79 (0.47–1.12)
Q3 (4.64, 5.79)	10.83	28,610	294,656.7	145	0.49 (0.24–0.75)
Q4 (5.79, 7.44)	9.33	28,610	296,603.7	171	0.58 (0.30–0.86)
Q5 (7.44, 52.65)	11.17	28,610	302,202.9	127	0.42 (0.18–0.66)

CVD, cardiovascular disease; CI, confidence interval.

**Table 3:** Multiple Cox proportional hazard regression analysis for all-cause mortality and cardiovascular disease (CVD) mortality of fiber intake quintiles.

	Q1	Q2	Q3	Q4	Q5
	(0.37, 3.51)	(3.51, 4.64)	(4.64, 5.79)	(5.79, 7.44)	(7.44, 52.65)
	Hazard ratios (95% Confidence intervals)				
All-cause mortality					
Unadjusted	1.00 (ref)	0.75	0.65	0.62	0.54
		(0.70, 0.81)	(0.60, 0.71)	(0.57, 0.67)	(0.50, 0.59)
Model 1	1.00 (ref)	0.89	0.83	0.85	0.84
		(0.82, 0.96)	(0.76, 0.90)	(0.78, 0.93)	(0.76, 0.94)
Model 2	1.00 (ref)	0.88	0.82	0.85	0.84
		(0.82, 0.96)	(0.76, 0.90)	(0.78, 0.93)	(0.76, 0.93)
CVD mortality					
Unadjusted	1.00 (ref)	0.75	0.47	0.55	0.39
		(0.64–0.89)	(0.38–0.57)	(0.45–0.66)	(0.32–0.48)
Model 1	1.00 (ref)	0.94	0.62	0.79	0.62
		(0.79–1.12)	(0.51–0.77)	(0.64–0.97)	(0.48–0.79)
Model 2	1.00 (ref)	0.93	0.62	0.78	0.61
		(0.78–1.10)	(0.50–0.76)	(0.64–0.96)	(0.47–0.78)

Model 1: adjusted for age, sex, body mass index, smoking, alcohol intake, exercise, and total calories. Model 2: adjusted for age, sex, body mass index, smoking, alcohol intake, exercise, total calories, hypertension, diabetes, and dyslipidemia

## Data Availability

The data used in this study were available on the following website: http://www.cdc.go.kr/contents.es?mid=a40504100100 (accessed on 27 December 2021).

## Supplementary Materials

The following are available online at https://www.mdpi.com/article/10.3390/nu14153089/s1. Table S1: Multiple Cox proportional hazard regression analysis for all-cause mortality and cardiovascular disease (CVD) mortality of fiber intake quintiles according to the presence of hypertension, diabetes, and dyslipidemia.
