# Supplementary material for: Association of Dietary Fiber Intake with All-Cause Mortality and Cardiovascular Disease Mortality: A 10-Year Prospective Cohort Study
Source: Nutrients. 2022 Jul 27;14(15):3089. doi: 10.3390/nu14153089 (PMC9370192; doi:10.3390/nu14153089)
Supplement: Supplementary file 1 [file nutrients-14-03089-s001.zip › nutrients-1823152-supplementary.pdf]

Supplementary Table S1. Multiple Cox proportional hazard regression analysis for all-cause mortality and cardiovascular disease (CVD) mortality of fiber intake quintiles according to the presence of hypertension, diabetes and dyslipidemia

**Hypertension**	Q1<br>(0.42, 3.48)	Q2<br>(3.48, 4.62)	Q3<br>(4.62, 5.77)	Q4<br>(5.77, 7.45)	Q5<br>(7.45, 41.7)
			Hazard ratios<br>(95% Confidence intervals)		
All-cause mortality					
Unadjusted	1.00 (ref)	0.79<br>(0.68, 0.91)	0.67<br>(0.58, 0.79)	0.62<br>(0.53, 0.73)	0.55<br>(0.47, 0.65)
Model 1	1.00 (ref)	0.85<br>(0.73, 0.99)	0.78<br>(0.67, 0.92)	0.76<br>(0.65, 0.89)	0.72<br>(0.61, 0.85)
Model 2	1.00 (ref)	0.87<br>(0.75, 1.02)	0.82<br>(0.69, 0.97)	0.80<br>(0.67, 0.95)	0.77<br>(0.63, 0.94)
Model 3*	1.00 (ref)	0.88<br>(0.75, 1.03)	0.84<br>(0.70, 0.99)	0.82<br>(0.68, 1.00)	0.79<br>(0.61, 1.03)
CVD mortality					
Unadjusted	1.00 (ref)	0.80<br>(0.59, 1.09)	0.57<br>(0.41, 0.80)	0.59<br>(0.43, 0.83)	0.55<br>(0.39, 0.77)
Model 1	1.00 (ref)	0.91<br>(0.66, 1.23)	0.71<br>(0.51, 1.01)	0.79<br>(0.56, 1.10)	0.79<br>(0.56, 1.11)
Model 2	1.00 (ref)	0.90<br>(0.66, 1.23)	0.70<br>(0.49, 1.00)	0.75<br>(0.52, 1.09)	0.72<br>(0.47, 1.09)
Model 3*	1.00 (ref)	0.92<br>(0.67, 1.27)	0.73<br>(0.51, 1.06)	0.81<br>(0.54, 1.21)	0.80<br>(0.47, 1.36)

**Diabetes**	Q1	Q2	Q3	Q4	Q5
	(0.59, 3.45)	(3.45, 4.53)	(4.53, 5.66)	(5.66, 7.32)	(7.32, 40.9)
		Hazard ratios (95% Confidence intervals)			
All-cause mortality					
Unadjusted	1.00 (ref)	0.78	0.69	0.70	0.53
		(0.64, 0.96)	(0.56, 0.85)	(0.57, 0.85)	(0.43, 0.66)
Model 1	1.00 (ref)	0.81	0.75	0.79	0.64
		(0.66, 0.99)	(0.61, 0.92)	(0.64, 0.97)	(0.51, 0.80)
Model 2	1.00 (ref)	0.84	0.83	0.90	0.77
		(0.69, 1.03)	(0.67, 1.03)	(0.72, 1.12)	(0.59, 1.00)
Model 3†	1.00 (ref)	0.84	0.82	0.90	0.76
		(0.68, 1.04)	(0.66, 1.03)	(0.70, 1.15)	(0.55, 1.07)
CVD mortality					
Unadjusted	1.00 (ref)	0.73	0.59	0.78	0.33
		(0.46, 1.15)	(0.36, 0.96)	(0.50, 1.21)	(0.19, 0.59)
Model 1	1.00 (ref)	0.77	0.65	0.91	0.42
		(0.49, 1.21)	(0.40, 1.06)	(0.58, 1.43)	(0.24, 0.74)
Model 2	1.00 (ref)	0.78	0.67	0.95	0.44
		(0.49, 1.24)	(0.40, 1.11)	(0.58, 1.57)	(0.22, 0.87)
Model 3†	1.00 (ref)	0.75	0.62	0.84	0.33
		(0.47, 1.20)	(0.36, 1.04)	(0.49, 1.45)	(0.14, 0.75)
**Dyslipidemia**	Q1	Q2	Q3	Q4	Q5
	(0.37, 3.51)	(3.51, 4.64)	(4.64, 5.79)	(5.79, 7.44)	(7.44, 52.65)

		Hazard ratios (95% Confidence intervals)			
All-cause mortality					
Unadjusted	1.00 (ref)	0.71	0.62	0.56	0.50
		(0.64, 0.79)	(0.56, 0.69)	(0.50, 0.63)	(0.49, 0.56)
Model 1	1.00 (ref)	0.80	0.71	0.69	0.67
		(0.72, 0.88)	(0.64, 0.79)	(0.62, 0.77)	(0.60, 0.75)
Model 2	1.00 (ref)	0.83	0.76	0.75	0.74
		(0.75, 0.92)	(0.68, 0.85)	(0.66, 0.85)	(0.65, 0.86)
Model 3‡	1.00 (ref)	0.84	0.78	0.78	0.79
		(0.75, 0.93)	(0.69, 0.87)	(0.68, 0.89)	(0.67, 0.95)
CVD mortality					
Unadjusted	1.00 (ref)	0.67	0.41	0.49	0.35
		(0.54, 0.84)	(0.32, 0.53)	(0.39, 0.62)	(0.27, 0.45)
Model 1	1.00 (ref)	0.79	0.51	0.66	0.51
		(0.64, 0.99)	(0.40, 0.66)	(0.52, 0.84)	(0.39, 0.67)
Model 2	1.00 (ref)	0.82	0.53	0.69	0.53
		(0.66, 1.03)	(0.41, 0.70)	(0.53, 0.91)	(0.38, 0.73)
Model 3‡	1.00 (ref)	0.83	0.56	0.73	0.57
		(0.66, 1.04)	(0.42, 0.74)	(0.54, 0.99)	(0.38, 0.87)

Model 1: adjusted for age, sex, and body mass index (BMI)
Model 2: adjusted for age, sex, BMI, smoking, alcohol intake, exercise, and total calorie
Model 3*: adjusted for age, sex, BMI, smoking, alcohol intake, exercise, total calorie, diabetes, and dyslipidemia
Model 3†: adjusted for age, sex, BMI, smoking, alcohol intake, exercise, total calorie, hypertension, and dyslipidemia
Model 3‡: adjusted for age, sex, BMI, smoking, alcohol intake, exercise, total calorie, hypertension, and diabetes
